# On the challenges associated with the study of police use of deadly force in the United States: A response to Schwartz & Jahn

**DOI:** 10.1371/journal.pone.0236158

**Published:** 2020-07-28

**Authors:** Justin Nix

**Affiliations:** School of Criminology and Criminal Justice, University of Nebraska Omaha, Omaha, Nebraska, United States of America; London School of Economics, UNITED KINGDOM

## Abstract

In response to Gabriel Schwartz and Jaquelyn Jahn’s descriptive study, “Mapping fatal police violence across U.S. metropolitan areas: Overall rates and racial/ethnic inequalities, 2013–2017,” I provide three reflections. First, the framing of this issue is vitally important. Second, police-involved fatalities represent a nonrandom sample of all incidents involving police use of deadly force (i.e., physical force that causes or is likely to cause death), and unfortunately, we lack comprehensive data on use of deadly force that does not result in fatalities. Finally, to make sense of who is killed by the police, researchers must also identify who was exposed to the risk of being killed by the police.

## Introduction

Gabriel Schwartz and Jaquelyn Jahn analyzed data from Fatal Encounters—the most comprehensive database tracking police-involved deaths in the United States—and uncovered vast differences across metropolitan statistical areas (MSAs) in the overall rates of persons killed by police officers, as well as racial inequities in those rates, between 2013 and 2017 [1]. The key contribution of their analysis is that it was conducted at the MSA level, which offers a more precise look at variation in police-involved fatalities across the United States than most prior work. Four findings were especially noteworthy. First, overall rates varied extensively, from a low of 0.13 police-involved fatalities per 100,000 in Buffalo-Cheektowaga-Niagara Falls, NY to 1.17 police-involved fatalities per 100,000 in Anniston-Oxford-Jacksonville, AL. Second, western MSAs generally experienced higher annual rates of police-involved fatalities than their counterparts in the northern Midwest and Northeast. Third, across 382 MSAs, the Black incidence rate of fatalities involving police was at least 1.81 times higher than the White incidence rate (though notably, the 95% confidence intervals were wide and the vast majority included 1.00). In the Chicago-Naperville-Elgin MSA, for example, the Black incidence rate of fatalities involving police was 6.51 times (95% CI 1.84, 23.09) higher than the White incidence rate. Finally, Latinx-White inequities were less pronounced than Black-White inequities. In the Pueblo, CO MSA—which had the most extreme Latinx-White inequity from 2013 to 2017 –the Latinx incidence rate of fatalities involving the police was 1.50 times higher than the White incidence rate.

Schwartz and Jahn performed a purely descriptive, demographic analysis. In other words, they were interested in documenting variation in police-involved fatality rates across MSAs, not attempting to explain said variation. That requires more data. As with any social phenomenon, there is value in understanding “what” before chasing answers to “why” and suggesting reforms. But as Chowkwanyun and Reed remind us, “disparity figures without explanatory context can perpetuate harmful myths and misunderstandings that actually undermine the goal of eliminating health inequities” [2]. As such, Schwartz and Jahn’s study must be interpreted with three critical details in mind. First, we must consider the way we frame these discussions. Second, although Fatal Encounters offers the most comprehensive data on police use of deadly force in the United States, it likely misses hundreds, if not thousands, of police uses of deadly force each year [3]. Finally, the authors’ use of census data to construct the denominator for each MSA’s police-involved fatality rate rests on a strong assumption: that every person residing in each MSA is exposed to the risk of being killed by a police officer. I expand on these issues in the following discussion.

## Framing

As researchers increasingly take advantage of datasets like Fatal Encounters, it is imperative that they inspect the data to ensure they are measuring what they intend to measure [4]. Accordingly, I applaud Schwartz and Jahn for excluding from their analysis 1,670 deaths that resulted from suicides, accidents, or vehicular collisions (in a supplemental analysis, they show what the results would be with these incidents included). However, as is common practice in public health and epidemiological research, the authors framed their study as one pertaining to *fatal police violence* [5–8], which they define as “fatalities in police custody or involving the police that would not have occurred in the absence of police intervention.” There is no disputing that when police kill, they do so via acts of physical violence. But labelling *every* police-involved death “fatal *police* violence” assigns all responsibility to officers, as if none of the citizens involved contributed in any way to the violence.

Criminologist Philip Stinson, an expert on police crime and integrity, defines police violence as “any amount of force…that cannot be accounted for under the auspices of lawful necessity in the line of duty” (p. 14) [9]. Following Stinson’s definition, fatal police violence is an appropriate way to describe the killings of Breonna Taylor, George Floyd, Walter Scott, Philando Castile, Eric Garner, Tamir Rice, and Amadou Diallo (to name just a few). It should not be used broadly to describe what criminologists have long referred to as *police use of deadly force* [10]. Unfortunately, its usage is likely to polarize and distract from Schwartz and Jahn’s important study.

Words matter. A vast literature on framing theory suggests they can have powerful effects on the way people “develop a particular conceptualization of an issue or reorient their thinking about an issue” [11; see also 12]. Recently, Fridkin et al. [13] conducted an experiment where 225 political science students viewed dashcam footage of the arrest of Ersula Ore, “an African American professor at a major southwestern university [who] was ‘body slammed’ to the ground…for jaywalking.” Prior to watching the footage, students were randomly assigned to read brief introductory paragraphs that framed the incident in terms of *law and order* (emphasizing the jaywalking violation, the officer’s concern for public safety, and the professor’s “violent outburst”), *police brutality* (emphasizing the body slam and charges being pressed against the officer for excessive force), or *race* (emphasizing the race of both the officer and the professor, and mentioning a civil rights investigation). A fourth control stimulus simply informed students that a professor’s arrest was “creating quite a stir,” and directed them to watch the footage. Unsurprisingly, despite the fact that all participants viewed the same video, exposure to the *law and order* and *police brutality* frames significantly affected students’ evaluations of both the officer and the professor. Framing of the event even appeared to indirectly influence their broader perceptions of racist policing being a problem in their community.

Now, consider the implications of framing all 5,494 police-involved fatalities that occurred from 2013 to 2017 as resulting from “police violence.” One of the victims of “police violence” in Schwartz and Jahn’s analysis is Salvador Reyes, killed on October 17, 2016 by a Tulsa (OK) police officer. This occurred following a three-hour standoff with Reyes, an estranged ex-husband who had broken into his ex-wife’s home, grabbed her two-year-old daughter, and held the girl at gunpoint on a balcony. Ultimately, a police sniper shot and killed Reyes, saving the little girl’s life (see S1-A of S1 Data). This was without question a *domestic violence* incident that ended with police using deadly force. Another victim of “police violence” in the sample was Micah Xavier, who ambushed Dallas (TX) police officers in July 2016, killing five and injuring nine others. Eventually, police killed Johnson with a bomb disposal robot (see S1-B of S1 Data). Though incidents like these should absolutely be documented and studied alongside other police uses of deadly force, it is debatable whether they (and many others) should be reframed as “police violence.”

Indeed, much (but not all) of the time when officers use deadly force, it follows or preempts a perceived imminent deadly threat—either to their own lives or the lives of other citizens. Although the Fatal Encounters dataset does not include a field indicating whether the decedent was armed, as of March 18, 2020, *The Washington Post* reports that roughly 87% of the 5,134 citizens fatally shot by police officers since 2015 were in possession of a potentially deadly weapon (i.e., a firearm, knife/cutting instrument, or blunt object). Most who had firearms posed a direct and immediate threat to officers, according to *The Washington Post* (see S1-C of S1 Data). Given the evidence regarding framing effects, use of the term “police violence” has the potential to mislead readers who believe that police use of deadly force is rampant and usually unjustified (e.g., those who view police as “vigilantes” or “oppressors”; see [14]). It also has the potential to drive away readers who understand how statistically rare police use of deadly force is, and that it usually occurs in *response* to violence (e.g., police officers themselves, and those who view police as “professionals”; see [14] and [15–22]). I suspect *police-involved fatalities* is less leading, but this is ultimately an empirical question that future research should consider.

## Generalizing from a nonrandom sample of deadly force incidents

The foremost cause of death at the hands of police, by far, is gunshot wounds. Ninety-four percent of the deaths in Schwartz and Jahn’s sample resulted from police gunfire. However, like other crowdsourced datasets tracking police use of deadly force, Fatal Encounters only documents police shootings that result in the death of a person. According to the late James Fyfe [23], a pioneer in the study of officer-involved shootings:

*The true frequency of police decisions to employ firearms as a means of deadly force*…*can best be determined by considering woundings and off-target shots as only fortuitous variations of fatal shootings*. *All are of a kind*”

*(p*. *32)*.

That is, each time a police officer points and shoots a firearm at a person, a deadly force incident has transpired—even if the shot(s) misses or the person survives.

In the absence of national-level data on nonfatal police shootings, we are left to estimate how often they occur. Studies conducted at lower levels of analysis (i.e., one or more agencies) suggest police shootings result in death anywhere from 15 to 50% of the time [3, 24–30]. The five years of Fatal Encounters data analyzed by Schwartz and Jahn include approximately 5,000 deaths resulting from police shootings. If we assumed that the police shooting fatality rate over this period was 50% (a liberal estimate), it would mean the authors’ analysis excluded 5,000 police shootings that did not result in death (but nevertheless qualify as police uses of deadly force).

Here is why this matters: the numerators of Schwartz and Jahn’s fatality rates are a *nonrandom sample* of all deadly force incidents that occurred from 2013 to 2017. To be sure, there is some degree of chance in whether a person who is shot lives or dies (e.g., whether bullets pierce a vital organ) [31–33]. But part of the variation across MSAs both in terms of rates of police-involved fatalities and racial disparities therein might be driven by nonrandom factors apart from police behavior. One such factor is trauma care accessibility. Proximity to trauma centers varies systematically across communities [34–36], and prior research indicates that shooting victims face an elevated risk of dying from their wounds when they are shot farther away from trauma-certified hospitals [37–40]. Schwartz and Jahn’s analysis does not tell us the extent that police use of deadly force varies across MSAs; instead, it tells us the extent that the police-involved fatality rate varies across MSAs. This is a crucial distinction.

Any two MSAs could be remarkably similar in terms of the rate at which officers use deadly force, yet quite different in terms of the rate at which people succumb to wounds inflicted by police officers. Or vice versa. According to *VICE News*, the Las Vegas Metropolitan Police Department (LVMPD) and the St. Louis Metropolitan Police Department (SLMPD) were involved in 115 and 119 police shootings, respectively, from 2010 to 2016 [3]. That is, these two departments were involved in roughly the same number of deadly force incidents. However, LVMPD’s fatality rate was 40.9% versus SLMPD’s 16.8%. Thus, 47 of LVMPD’s shootings were fatal, compared to just 20 of SLMPD’s shootings. An analysis like Schwartz and Jahn’s, conducted with these two cities, would lead to the mistaken conclusion that fatal police violence was twice as common in Las Vegas as St. Louis, despite officers in these departments using deadly force on a similar number of occasions. Meanwhile, whereas the Boston and Atlanta Police Departments were involved in 10 fatal police shootings each, Boston had just 4 additional nonfatal shootings (fatality rate = 71.4%), while Atlanta had an additional 32 (fatality rate = 23.8%) [41]. Here, an analysis like Schwartz and Jahn’s would lead to the conclusion that fatal police violence occurred with the same frequency in each city, when in fact Atlanta officers were involved in 3x as many deadly force incidents. Descriptive studies limited primarily to police shootings that result in the death of a person, instead of all occasions in which officers used deadly force, can lead to mistaken inferences about racial disparities in the rate at which the police use deadly force [42].

To be clear, the lack of national data on nonfatal police shootings is a frustrating limitation with which we all must grapple. As researchers, we can either (1) abandon the study of national trends in police use of deadly force and focus on smaller units of analysis [43, 44] or (2) own the limitations of the data and be transparent about what can and cannot be concluded from analyses of them. Schwartz and Jahn’s analysis cannot tell us whether officers disproportionately use deadly force against Black citizens, nor whether officers in western MSAs use deadly force at a significantly higher rate than their counterparts in northeastern MSAs. Instead, their analysis tells us that overall and race-specific fatality rates vary across MSAs. To identify the reasons for this variation, we need more data.

## The challenge of defining the at-risk population

In trying to make sense of trends and patterns in some observable police behavior, such as the use of deadly force, researchers face the difficult task of defining and measuring the counterfactual [45]. In Schwartz and Jahn’s case, this would be the population of individuals who interacted with a police officer from 2013 to 2017 but were not killed. Everyone in this population was exposed to the risk, however small, of being killed by a police officer [46]. However, Schwartz and Jahn calculated their fatality rates with census data—meaning they included in their denominators millions of people who did not interact with a police officer, and whose risk of being killed by a police officer was approximately 0% (see S1-D of S1 Data). Data collected by the Bureau of Justice Statistics suggest only about 21% of the 253.6 million US residents age 16 or older had some sort of contact with a police officer in 2015 (i.e., a police officer stopped them, they were involved in traffic accident, or they called the police) [47]. Among those stopped each year, less than 2% are threatened with or subjected to police use of force [48]. One can only be subjected to police force—including deadly force—conditional on interacting with a police officer in time and space. So how informative is it to calculate police-involved fatality rates for a population that is mostly never at risk?

The authors defend their methodological decision as follows:

*We use population denominators to align with*, *and allow comparisons to*, *previous demographic work in this area*, *and because using race-specific crime or arrest counts—themselves shaped by racial bias and segregation—yields estimates of a different and potentially biased contrast than the rather simple ones we answer here*…

The authors are correct in their assertion that race-specific crime or arrest benchmarks are flawed. Meta-analytic studies have documented clear racial disparities in arrests [49], and furthermore, the available data demonstrate that deadly force incidents do not occur exclusively during the investigation of criminal activity or in the course of making arrests [45]. In other words, being killed by a police officer is not conditional on committing a crime or being arrested.

The authors continue:

…*in which metropolitan areas are people most likely to be killed by police*, *what is the difference in these rates by race*, *and how does this vary across MSAs*?

Answering these questions requires knowing how many people in each MSA *were not killed by police*. Knowing how many were not killed arguably requires determining who was *at risk* of being killed. A Venn diagram of the “at risk” and general populations would not perfectly align—instead, the “at risk” circle would be a small circle within the much larger general population circle. Perhaps some comparisons to other phenomena are in order. To estimate maternal mortality rates, researchers do not include all women in the denominator, but instead the number of live births [50]. To estimate meaningfully the rate at which sexually transmitted diseases proliferate, researchers would want to identify the sexually active population [51]. Finally, to estimate the rate at which sharks bite people, researchers would need to determine who goes into the water [52]. Studies concerned with police use of deadly force must be equally attentive to identifying a *meaningful* denominator.

My stance is that by calculating police-involved fatality rates with police-citizen interactions in the denominator, we can at least be certain that everyone in the sample we are analyzing was exposed to some risk of being subjected to deadly force. This is no easy task, as reliable data on the rate at which police officers interact with people are not systematically collected. The Police-Public Contact Survey is administered in three-year intervals, and provides only a national-level snapshot, which precludes investigating the substantial variation that occurs at smaller units of analysis, as Schwartz and Jahn did. Further complicating matters, being stopped by police officers may be a mediator on the causal pathway between race and police use of deadly force, and the available evidence indicates that both crime reporting [53–55] and proactive police stops [56–59] differ systematically across racial groups. As such, conditioning on stops could bias analyses by (1) blocking a mediating path and (2) inducing collider stratification bias [58, 60]. Is the solution to ignore this mediator—which is literally a necessary precondition for being killed by a police officer—and calculate rates for the entire population (most of whom are never at risk)? If the goal is to understand and improve officer decision-making as a way to save lives, then I am not convinced. Stopping a person and using deadly force on a person are two different decision points, with different antecedents, and need to be analyzed as such.

To be clear, there is nothing inherently wrong about Schwartz and Jahn’s use of population denominators in their analysis, so long as readers bear in mind there are *many* factors (including police behavior) that drive the disparities [53–67]. I am merely pointing out that it produces rates that are not all that helpful in understanding *why* police-involved fatalities vary across space as they do. But again, in fairness, the authors made no attempt to explain the underlying reasons for said variation. Future research must do so.

## Where to go from here?

I believe a lot can be gleaned from Schwartz and Jahn’s article, and agree that “place-specific policy contexts are likely a major cause of the distribution of overall incidence rates.” The authors specifically mention “state and local firearm regulations, levels of segregation and policy drivers of those levels, or differences in police training and police department protocols.” Daniel Nagin, for example, uncovered a “correlation between statewide prevalence of gun ownership and fatal police shootings for both all decedents and unarmed decedents,” and confirmed that “greater access to trauma centers is associated with lower rates of citizen deaths” [68]. In a separate study, Jay Jennings and Meghan Rubado showed that agencies requiring officers to file a report when they point their firearms at people experience significantly lower rates of fatal police shootings [69]. In short, reducing police-involved fatalities requires a multi-faceted approach.

From the police perspective, it will require reducing the frequency with which officers are forced to make “split-second decisions.” In part, this means getting officers to slow down and reduce their sense of urgency [70]. As Lawrence Sherman [71] notes:

*[C]onventional viewpoint places the policy for when to shoot in the context of the split second when an officer pulls the trigger—thereby ignoring all the contextual factors that shape (and limit) the choices of any officer who arrives at that split-second*, *final frame*. *Viewing a police encounter as a movie*, *we can rewind the movie to identify many previous* “*frames*” *in the reel of film*, *in which a different choice may have saved everyone from harm*

(p. 11).

Improved decision making in the earlier “frames” of police-citizen interactions might be achieved via training [72–74] and policy [75, 76], but Philip Atiba Goff and Hilary Rau argue that agencies would get more return on investment if they focused on how they screen applicants [77]. There are, of course, more drastic measures that could be taken. In the wake of the recent police killings of Breonna Taylor and George Floyd (and ongoing protests across the world), there is currently a push to defund local police departments and rethink their function in US society [78–82]. As communities explore these and other ways to reduce police-involved fatalities, it is imperative that they set clear and manageable goals, define at the onset what they would consider *success* or *failure*, and rigorously evaluate the effects of the policies or interventions implemented.

As important, our understanding of police use of deadly force continues to be hindered by a lack of comprehensive, national data. Fortunately, some states have begun collecting more comprehensive use of force data from their respective local agencies (see “Police Involved Deaths and Use of Force” at https://www.ncsl.org/research/civil-and-criminal-justice/law-enforcement.aspx#3). Andrew Wheeler and colleagues recently proposed that the Federal Bureau of Investigation (FBI) add fields to its National Incident-Based Reporting System (NIBRS) [44]. Specifically, agencies could provide information about officer-involved shootings (fatal and nonfatal) as well as less-lethal forms of force, including weapon draws, Taser uses, and empty-hand tactics. This would allow researchers to analyze use of force incidents alongside those that do not result in force [83], and would make for more informative comparisons of trends across time and space. The FBI plans to transition fully to NIBRS by 2021, so participation by state and local agencies should increase significantly in the coming years. The transition could be a golden opportunity to collect better data.

Improved data will enhance researchers’ ability to describe *how* use of deadly force varies across jurisdictions, as well as to explore the viable reasons *why* it varies the way it does. Ultimately, the answers to these questions will help us identify plausible ways to save lives.

## Supporting information

S1 Data (DOCX)
