# Supplementary material for: On the challenges associated with the study of police use of deadly force in the United States: A response to Schwartz & Jahn
Source: PLoS One. 2020 Jul 28;15(7):e0236158. doi: 10.1371/journal.pone.0236158 (PMC7386827; doi:10.1371/journal.pone.0236158)
Supplement: S1 Data — (DOCX) [file pone.0236158.s001.docx]

**Supporting Information**

A. See <https://www.washingtonpost.com/news/post-nation/wp/2016/10/24/an-armed-man-held-a-toddler-hostage-for-hours-a-police-sniper-fired-one-shot-and-saved-her/>.

B. See <https://www.washingtonpost.com/news/morning-mix/wp/2016/07/08/like-a-little-war-snipers-shoot-11-police-officers-during-dallas-protest-march-killing-five/>.

C. See “About the Data” at <https://github.com/washingtonpost/data-police-shootings>. The majority of individuals who possessed a firearm are classified as “attack” in *The Washington Post’s* “threat_level” field.

D. *Approximately,* because much of the population does in fact have a non-zero risk of being killed by police officers. Vehicular pursuits routinely result in deaths of innocent drivers, passengers, and bystanders (see, e.g., <https://www.usatoday.com/story/news/2015/07/30/police-pursuits-fatal-injuries/30187827/>). But recall that Schwartz and Jahn excluded these non-intentional killings from the numerators in their primary analyses.
